# How Heavy Lifting Lightens Our Lives: Content Analysis of Perceived Outcomes of Masters Weightlifting

**DOI:** 10.3389/fspor.2022.778491

**Published:** 2022-03-15

**Authors:** Marianne Huebner, Holly Arrow, Alex Garinther, David E. Meltzer

**Affiliations:** ^1^Department of Statistics and Probability, Michigan State University, East Lansing, MI, United States; ^2^Department of Psychology, University of Oregon, Eugene, OR, United States; ^3^College of Integrative Sciences and Arts, Arizona State University, Mesa, AZ, United States

**Keywords:** weightlifting, competition, gender differences, psychosocial benefits, sport, aging, health

## Abstract

Although the benefits of sport participation for older adults has been well-documented, the traditionally masculine sport of weightlifting has only recently become popular among older women, who now participate at rates comparable to men in the United States. This study describes the self-reported effects of participating in Masters-level Olympic weightlifting on other aspects of life. Contrasting with previous studies of Masters athletes in other sports, the gender balance and broad age range of our sample allowed us to explore whether the self-reported impact of sport on older adults was similar or different across age groups (35–44, 45–59, 60, and older) for both men and women. A total of 352 (191 women, 159 men, 2 other) who completed a survey of Masters lifters registered with the United States national organization (USAW) responded to an open-ended question about how weightlifting has affected other aspects of their life. Across gender and age categories, responses indicated that weightlifting has a positive impact on physical health (strength, mobility, fitness) and on psychological (mental health benefits, stress reduction) and social aspects such as community connections. Female lifters mentioned psychological benefits such as increased confidence and help with stress and depression more commonly than male lifters; older lifters were more likely than middle-aged lifters to mention physical health benefits. Competition was a prominent theme across genders and age groups. The themes mentioned by participants are consistent with previous literature on sports that are less strongly gender-typed than weightlifting.

## Introduction

*The joy of weightlifting has given me purpose and motivated me to live an athlete's lifestyle; watching my diet, sleep, and training in a disciplined manner to achieve performance goals*. (Male, 45+)

*The effects have been only positive, physically, mentally, and emotionally. The best decision I ever made was to take up this sport and to be an active competitor!* (Female, 70+)

As the biomedical benefits of regular physical activity for aging adults have become clear (Galloway and Jokl, 2000; Tayrose et al., 2015), including slowing some of the processes associated with aging (Sun et al., 2013), calls for older adults to become more active have proliferated. The guidelines set by the World Health Organization include strength training at least twice a week for adults ages 18- 64 and three times per week for 65 and older (Bull et al., 2020). Engaging in strength-based physical activity to improve health differs from strength sports, which include a set of rules and require regular training to achieve defined goals. Different types of strength sports are weightlifting, powerlifting, body building, or throwing disciplines in track and field (Lavallee and Balam, 2010).

There is a paucity of research on experiences of older athletes in strength sports. Such research may contribute to insights about approaches to encourage appropriate strength programs for older populations. The purpose of the current study is to explore and describe the self-reported impact of training and competing in Masters level Olympic-style weightlifting on other aspects of life. Our study is based on responses of Masters-age members of the USA weightlifting organization (USAW) to a survey question asking, “*Do you have any comments how your weightlifting career affects other parts of your life?”* This impact may differ for male and female athletes and at different ages.

The Masters sports movement emerged in the last few decades of the twentieth century and has expanded since then. The most well-known early Masters events were in Athletics (track and field, running, and race-walking events) with the first World Masters Athletics Championships held in 1975. Ten years later came the first World Masters Games (WMG), which included 22 sports; Masters swimming held a separate championship (FINA World Masters) the following year [see (Young et al., 2015) for the early history of Masters sports]. The sport of Masters weightlifting, which is the focus of the current study, held its first World Masters Weightlifting Championships in 1985, for male lifters only, and the first Women's World Weightlifting Championships occurred 2 years later. It is a dynamic strength and power sport that demands considerable mobility and explosive force production (Storey and Smith, 2012). Weightlifters perform two competition lifts by rapidly moving heavy barbells from the ground to a locked-out position overhead, either in a single unbroken lift (the snatch) or in two steps, with a stop at the shoulders (the clean and jerk). For most of its history weightlifting was a sport for young men, and until the twenty-first century, Masters weightlifters were a small group, and overwhelmingly male. That has changed. In 2000, the same year that younger women weightlifters competed for the first time at the Olympic Games in Sydney, there were 382 athletes in the World Masters Weightlifting Championships, 13% of whom were women. Since then Masters women's participation has expanded rapidly, with 201women (25%) competing at the World Championships in 2016, and 316 (46%) in 2019 (https://www.iwfmasters.org/) (Huebner et al., 2021b). Masters men and women compete in 5-year age groups and in multiple body weight categories to provide fair chances in competitions for aging athletes.

By studying Masters weightlifters we broaden the general understanding of what “physical activity” can entail in middle and old age, contributing to the ongoing revision of cultural perceptions of aging (Baltes and Baltes, 1990). Interventions aimed at increasing physical activity have demonstrated limited success in promoting a long-term change in habits (Spence and Lee, 2003). Masters athletes show the kind of dedication toward regular training that ensures persistence.

Much of the work on older athletes has interviewed convenience samples of World Master Games participants from a mixture of mostly individual sports such as swimming or athletics (e.g., Dionigi et al., 2011). Sport-specific studies have included teams sports such as softball (Liechty et al., 2017; Naar et al., 2017), volleyball (Kirby and Kluge, 2013), and cricket (Jenkin et al., 2018). Our study expands on the coverage of individual Masters athletes in an exploratory study of impacts reported by Masters-aged USAW members, ages 35 to 85+, who train and compete in the individual sport of Olympic-style weightlifting. The sample includes a balance of weightlifters who identify as male and female plus two weightlifters with a gender identity of other or transgender.

The new ideal of lifelong physical activity as essential corresponds to new perspectives on aging, termed “successful aging” (Baltes and Baltes, 1990) or “positive aging” (Gergen and Gergen, 2001). These perspectives challenged the dominant narrative of aging as a process of decay, decline, and disability and suggested that the later years could also be characterized by active engagement in life, meaningful relationships, active physical activity, and psychological health. Early empirical studies inspired by this perspective were primarily guided by a sports-science and exercise-psychology perspective, and focused primarily on health and fitness benefits [see (Cousins, 2000) for an extensive review]. As Dionigi noted in his call for more qualitative studies (Dionigi, 2006), this early work rarely considered the sociocultural context or the participants' own views of aging and exercise and sport. Spence and Lee (2003), in noting the unimpressive history of interventions focused on the individual, also called for a broader perspective that considers multiple levels, including social and cultural contexts.

Following Spence and Lee's (2003) endorsement of a multi-level approach and Dionigi's exhortation to attend to the views of athletes, we draw on the socio-ecological framework (King and King, 2010) in our study and focus on athletes' reports of the benefits they experience from weightlifting across the span of ages from 35 to 85+. The socio-ecological framework is based on Bronfenbrenner's bioecological theory (Tudge et al., 2009) of human development as a process that unfolds over time through bidirectional influences between a person and their environment, making it a good match for the study of sport participation across the lifespan, including young athletes (Craike et al., 2009) and Masters athletes in their 50s, 60s, and 70s (Kirby and Kluge, 2013; Naar et al., 2017). In line with the bidirectional influences that are a core conceptual feature of ecological models (Tudge et al., 2009), the literature on Masters participation in competitive sports (see Cannella et al., 2021 for a recent review) indicates that the reasons for participating often coincide with the benefits that older athletes report. Hence our study of the perceived benefits and impacts reported by Masters-age weightlifters is relevant to understanding what might be responsible for a “virtuous circle” that promotes the persistence of older athletes in continuing to train and compete. It also provides a window on how different environmental systems levels considered (in intervention studies) as influences that might promote or discourage sport participation may themselves also be affected by Masters athletes.

King and King identified five components of the socio-ecological model (King and King, 2010; Naar et al., 2017). These are (1) personal characteristics such as sex and age; (2) individual behavior; (3) social/cultural elements such as interpersonal support, social norms, and cultural values; (4) environment/policy elements such as physical infrastructure and community programs; and (5) life course time effects.

In this study, we treat the personal characteristics (the first component of the socio-ecological model) of gender and age as potential influences on the self-reported outcomes of weightlifting. Our sample is unusual in the range of ages included, as most studies of Masters sport focus on athletes in the older age ranges; reviews of this literature typically use age cutoffs of 50+ (Kim et al., 2020; Cannella et al., 2021) 55+ (Stenner et al., 2020) or 65+ (Gayman et al., 2017). At the individual behavior level (the second component of the socio-ecological model), the demands of training and competing may affect other types of individual behavior such as health habits. Because lifters compete in weight classes, for example, nutritional choices are important not only for fueling effective training but also in managing bodyweight. The third, social/cultural component of the socio-ecological framework covers social benefits such as enhanced social support and the opportunity to meet new people (Grant, 2001; Kirby and Kluge, 2013), and a strengthened sense of community (Lyons and Dionigi, 2007).

Prior studies of Masters athletes that attend to the social/cultural systems level of social norms and cultural values regarding aging, gender, and competition highlight bidirectional influences. Athletes are both influenced by and actively push back against ageism (Jin and Harvey, 2020) and (for female athletes) gender norms (Ellemers, 2018). They may also experience social conflict with family members and friends (a “microsystem” in the socio-ecological model) who judge Masters athletes to be behaving inappropriately, e.g., “people saying that at your age shouldn't being doing this or that” (male in his 70s, Grant, 2001). We address the most macro socio-ecological level of environment/policy issues (the fourth component of the socio-ecological model) in considering the implications of our findings, and although our study is cross-sectional, the range of ages covered provides a basis for suggesting possible life course effects (the fifth component).

Prior studies also provide some indication of gender moderating self-reported outcomes for Masters athletes. In a study of men and women of comparable age and type of sports involvement, Eman reported that men, more than women, tended to find age-related declines in performance to be particularly discouraging, and viewed their sporting activities as a way to help slow the process of decline (Eman, 2012). In contrast, women tended to focus on their capabilities, and see themselves as “members of an empowered aging collective” (Eman, 2012). In a paired set of studies looking at social comparisons made by female (Horton et al., 2018) and male (Horton et al., 2019) WMG athletes, the men tended to rely on “negative role models” of less healthy, less active age peers as a way to “bolster their own psychological mindset and sense of self” (Horton et al., 2019), while the women tended to view themselves as positive role models, and hoped that by sharing their passion with friends and other age peers they “would inspire others to become more active” (Horton et al., 2018). In a quantitative study of Masters swimmers, men were significantly more likely than women to mention recognition for competitive achievement, while women were more likely to endorse stress relief, social affiliation, and opportunities for testing and self-assessment (Young et al., 2015). An important context for considering possible differences between the experiences of male and female Masters athletes is that many older women have experienced explicitly gendered reactions to their sport participation since they were young: “*When I was in school, there really wasn't much for girls… I guess there were people who thought athletic girls were kind of strange”* (female 70+, Horton et al., 2018).

Comparisons across different age groups within the span of Masters-aged athletes are rare: The study by Young and colleagues is a notable exception, as it included swimmers aged 25 to 70+, and tested for differences among four age ranges (Young et al., 2015). Although stress relief was mentioned by all groups, it was most prominent in the youngest group of age 25–39, less important in the 55–69 age group, and least important for athletes over 70. The opportunity to delay or counter aging (both the physical process and negative stereotypes) showed the opposite pattern, becoming more important with each increasingly older age group. The opportunity to test and assess one's abilities was valued most highly by the under-40 Masters. Qualitative studies have sometimes engaged the theme of life course effects *via* retrospective comparisons made by the athletes interviewed. Senior-aged female softball players (aged 55–79) who had also competed in their younger years reported that while senior ball was still competitive, the importance of winning as an outcome was less prominent (Naar et al., 2017).

With the broad age range covered and increasing gender parity, Masters weightlifting in the United States now provides a substantial pool of older athletes to study. This is an exploratory study using content analysis to identify potential gender and age range effects for a sport in which absolute performance declines reliably over age but follows a different pattern for men and women (Huebner et al., 2019).

## Materials and Methods

This is a primary data collection for a cross-sectional study of Masters age weightlifters based on content analysis of open-ended responses to an online survey.

### Study Population

All 3,216 Masters weightlifters registered with USA Weightlifting (USAW) during 2020 were invited to participate in this online survey. This group included all who would turn age 35 or older between January 1 and December 31 of 2020. The response rate was 30.6%. A study flow chart with exclusion criteria is provided in Supplementary Figure 1. A total of 352 Masters age weightlifters (191 women, 159 men, 2 other) are included in this study. The majority of respondents (98.1%) provided information about having competed in weightlifting. In addition to all survey questions, the study sample provided answers to an open-ended question about the impact of weightlifting on other aspects of life, which provides the data for the current study. The age range was from 34 to 87 (median 49) (Supplementary Table 1). The participants were primarily White/Caucasian (87.6%), Hispanics accounted for 7.7%. The education level was high, 31.6% had a college degree and a further 56.2% had a graduate degree.

### Procedures

The data for this study consists of responses to the following open-ended question “Do you have any comments about how your weightlifting career affects other parts of your life?” This question was included in a larger survey with closed-form questions about training habits, lifestyle, and health. The questionnaire was created and hosted in Qualtrics software, and an invitation to participate was sent *via* USAW's email system in January 2020 with a reminder 2 weeks later. Announcements with a link to the survey were also posted on Masters weightlifting Facebook sites. The larger survey was primarily designed to collect information on demographics, physical activities, training habits, health, and injuries, and how these affected participants' weightlifting training and competition. Consent was obtained and the study was approved by the Institutional Review Board of Michigan State University (Study No. 00003824). A total of 961 participants (response rate 30%) completed the larger survey that yielded results for the closed-form questions (Huebner et al., 2020).

Consistent with traditional approaches to content analysis (Malterud, 2001; Cho and Lee, 2014), a file with subject ID and answers to the target question was generated so that co-author HA could read through the responses and develop coding categories with no access to demographic information beyond what was revealed in the content of the comments themselves. Coauthor AG served as a second coder to establish reliability. After completion of the content coding a separate file was generated with answers to the target question that included gender, age, and age categories to be used for computer coding using the LIWC (Linguistic Inquiry and Word Count) system (Pennebaker et al., 2015) and the TagCrowd software (https://tagcrowd.com/) to generate word clouds.

The choice of age categories was based on the 5-year age groups in which Masters weightlifters compete, and distinguishes the younger range that includes age groups 35–39 and 40–44, which have the fastest-growing participation levels, a midlife category aged 45–59 (age groups 45–49, 50–54, and 55–59); and older athletes aged 60 or older (age groups 60–64 and up).

### Data Analysis

Categorical variables were summarized with frequency and percent; continuous variables were summarized with median and range. Multivariable linear models were used to study associations with the computer coded data (LIWC). Since the content coding were binary variables (presence of the topic), we used logistic regression models to estimate the probability of the outcomes based on age and gender. Outcome variables were proportions of words in the LIWC library corresponding to affective, social, and biological processes, drives, and work. Outcome variables for the qualitative content coding are described below. Explanatory variables were gender and (continuous) age in 5-year increments. Chi-square tests were used to compare groups for categorical variables, gender, and three broad age categories. The statistical models were based on 350 participants who identified as male or female, but all 352 were included in the coding. Since the demographics of survey participants who provided open ended comments differed from non-respondents, we also conducted a weighted regression analysis to balance factors associated with non-response (Hernán and Robins, 2006). *P* < 0.05 was considered statistically significant. Statistical analyses were performed using the statistical software R v.4.0.3 (R Core Team, 2021). The study was reported according to the Reporting Standards for Quantitative Research in Psychology (Appelbaum et al., 2018).

### Qualitative Content Coding

In defining categories, the goal was to generate no more than a dozen broad categories that would capture all responses. Reviews of the existing literature on the impact of sport for older athletes suggested some categories that would likely be important, such as effects on physical health/fitness, psychological health, and sense of community (e.g., Kim et al., 2020; Stenner et al., 2020). Other categories were generated to create a comprehensive coding system as the comments were reviewed. After a few iterations in which coauthors HA and AG tested the system by coding a trial set of 25 comments independently and then comparing and discussing results, the coding manual was simplified into 11 categories. The two coders used this manual to code an additional 47 responses independently, generating a total of 104 codes. Of these, 85 (81.7%) matched, which we judged to be acceptable reliability. After again discussing points of disagreement to resolution, coauthor HA coded the remaining 280 comments. The 10 categories were: 1. Overall physical health/fitness (*keeps me healthy*); 2. Specific health/medical issues, not including menopause/pregnancy (*my arthritis bothers me less*); 3. Reproductive health (impact on menopausal symptoms, pregnancy); 4. Impact on lifestyle / habits (such as reduced/eliminated smoking/drinking, attain/maintain health bodyweight); 5. Psychological (reduced anxiety/stress, improve mental toughness); 6. Meaning in life: Purpose/Goals/Identity (including competition); 7. Family/Community/Social (*makes me a better spouse; it's my social life*); 8. Requires (non-work-related) commitments (including time commitment, financial commitment, traveling); 9. Impact on work; 10. Other comments not clearly responsive to the question (these were often details about a lifter's personal athletic history).

### Computer Coding

LIWC analyzes text based on classifying words using an extensive library, and generates ratings on characteristics such as negative and positive emotion, social processes (including family, friends) biological processes (including health), psychological drives (including achievement, risk, and reward), and personal concerns (including work, leisure, money) based on the relative frequency of words that fit the LIWC library for these characteristics (Pennebaker et al., 2015). We chose the following 8 LIWC categories and subcategories to investigate: Affective processes plus the subcategories of positive and negative emotion; Social processes; Biological processes plus the health subcategory; Drives; Work (which is a subcategory of “Personal Concerns”).

### Word Clouds

Several word clouds were constructed based on participants' responses, overall and separately for women, men, and three age groups. These serve as a visual aid to complement the formal analyses based on the coding methods. Word clouds can be helpful for quickly identifying key concepts (DePaolo and Wilkinson, 2014). TagCrowd (https://tagcrowd.com/) displays the relative frequency of individual words in text through a “word cloud,” with more common words shown in larger font text. The process allows one to exclude “stop words,” which are typically common words that are not especially informative due to little variability (e.g., “weightlifting” in the current study). We designated the following stopwords: weightlifting, lifting, lift, lifter, train, training, and life. The first six words are all variants of the main topic of weightlifting and/or training. The word “life” appeared prominently across all groups in our preliminary clouds and is also related to the question prompt about how weightlifting affects one's life. To focus the attention on the main similarities and differences between gender and age groups, we limited the number of words to 16 per cloud and eliminated words that appeared 3 or fewer times. We also used the “group similar words” function so that, for example, instances of “help/helps/helping” would be counted together and collapsed into a single word. To parallel the other analyses, we generated separate clouds for words generated by women and by men, and for the three age categories collapsed across gender.

## Results

The median age for the current sample was 49 (range 34–87). Racial/ethnic composition was primarily white/Caucasian (88%); among other identities the most prevalent were Asian (4%), Black/African American (2%), Native Hawaiian/Pacific Islander (1%) other and various combinations of ethnicities (5%). In addition, 8% reported a Hispanic background. The sample was highly educated: over 80% had college degrees and 56% had attended graduate school. Only 1% (4 participants) listed high school or GED as their highest level of education. The sample was also highly affluent: a majority reported annual incomes over $100K.

Compared to the 609 weightlifters (330 women, 278 men, 1 other) who completed the larger survey but did not provide responses to the target question, our study participants were older (median age was 49 of respondents compared to 44 of non-respondents) with a higher proportion of white participants (88 vs. 82% for respondent and non-respondents, respectively). The racial/ethnic distribution and education levels were very similar to those of non-respondents (Supplementary Table 1). After applying case weights, the demographics between these two groups of participants are comparable (Supplementary Table 2).

### Qualitative Content Coding

**Figure 1:**
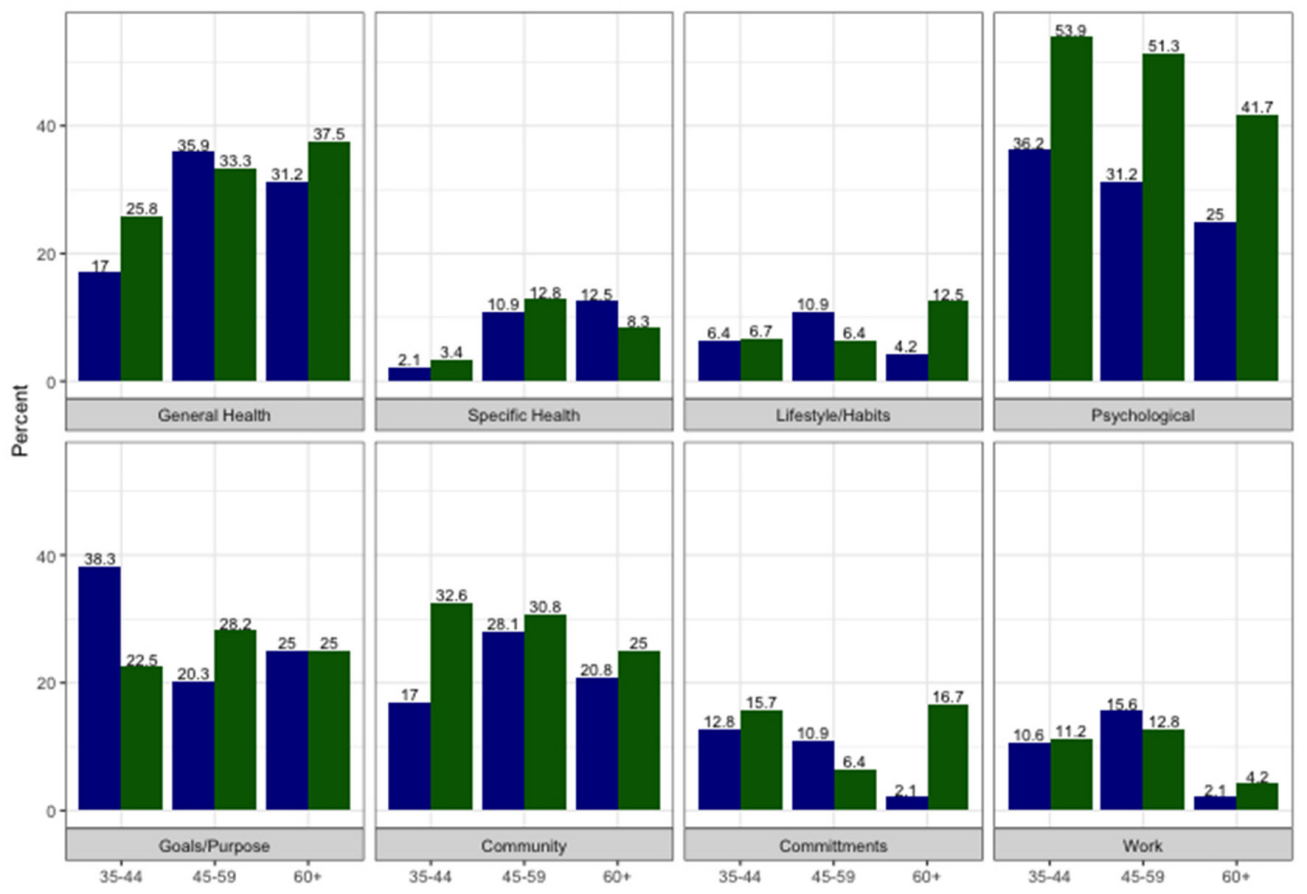
Percent of comments coded in different content categories by gender and age groups (blue for men and green for women).

The categories in the content analysis with the highest frequency comments were psychological (42%) overall physical health/fitness (29.7%), community/family (27.1%) and goals/meaning in life (26.0% total) (Figure 1). Overall frequencies for the other categories above 10% were commitments required (non-work) and work (10.6% for each). Comments were sparser in the categories of specific health (8.2%), lifestyle/habits (7.4%), and reproductive health (0.9%). The overwhelming majority of responses noted positive impacts of weightlifting; 22 responses (6.2%) also included comments about negative impacts, and the majority of these were from women (*n* = 16). Most were coded into the “commitment required” category and addressed the stress of time commitments and conflicts with family, work, or relationship demands.

### Computer Coding

**Figure 2:**
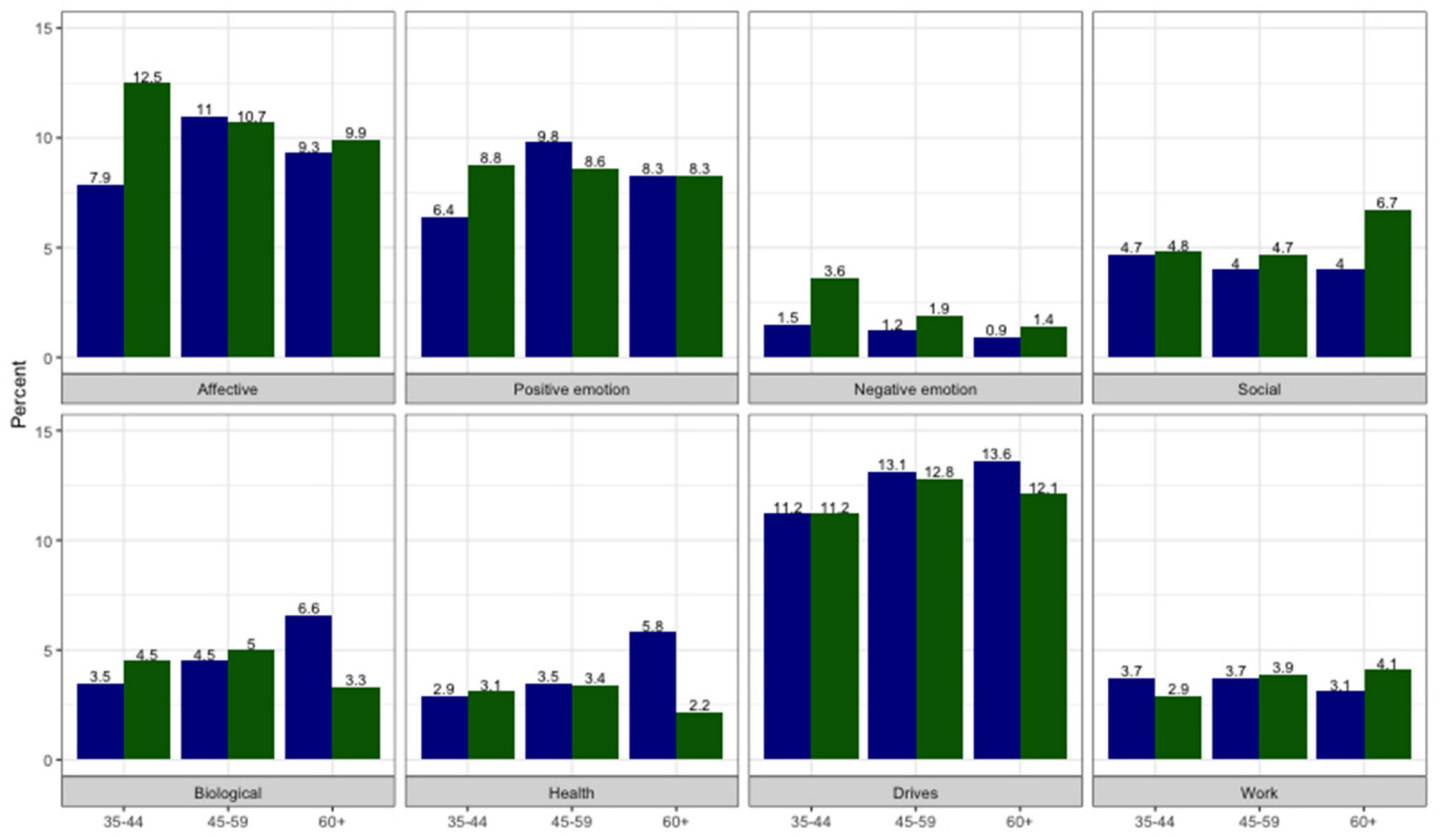
Percent of words classified in different content categories by LIWC libraries by gender and age groups (blue for men and green for women).

**Figure 3:**
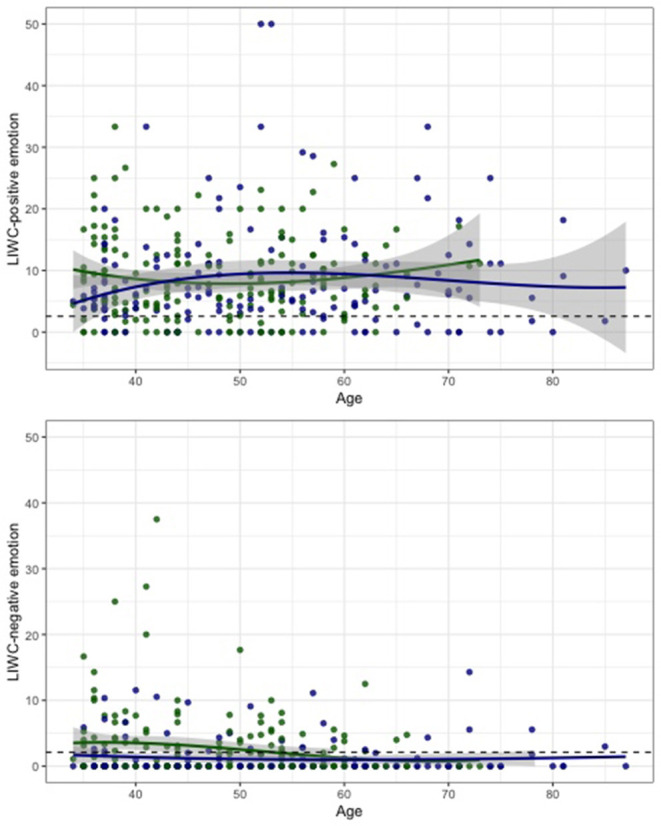
Percent of words classified as LIWC negative and positive emotion by age with cubic splines and confidence intervals (gray shaded areas). The overall mean from expressive writing is included as a reference line (dashed).

In LIWC, affective processes, such as positive and negative emotions, anxiety, anger (10.6%), and drives, such as affiliation, achievement, reward (12.3%), were categories with the highest frequency of words (Figure 2). The predominance of positive comments noted in the content coding was also picked up by the LIWC for positive emotion, as is indicated by the smoothed curves with confidence intervals which are above the mean for expressive writing from individuals from all walks of life (Figure 3) (Pennebaker et al., 2015).

### Word Clouds

**Figure 4:**
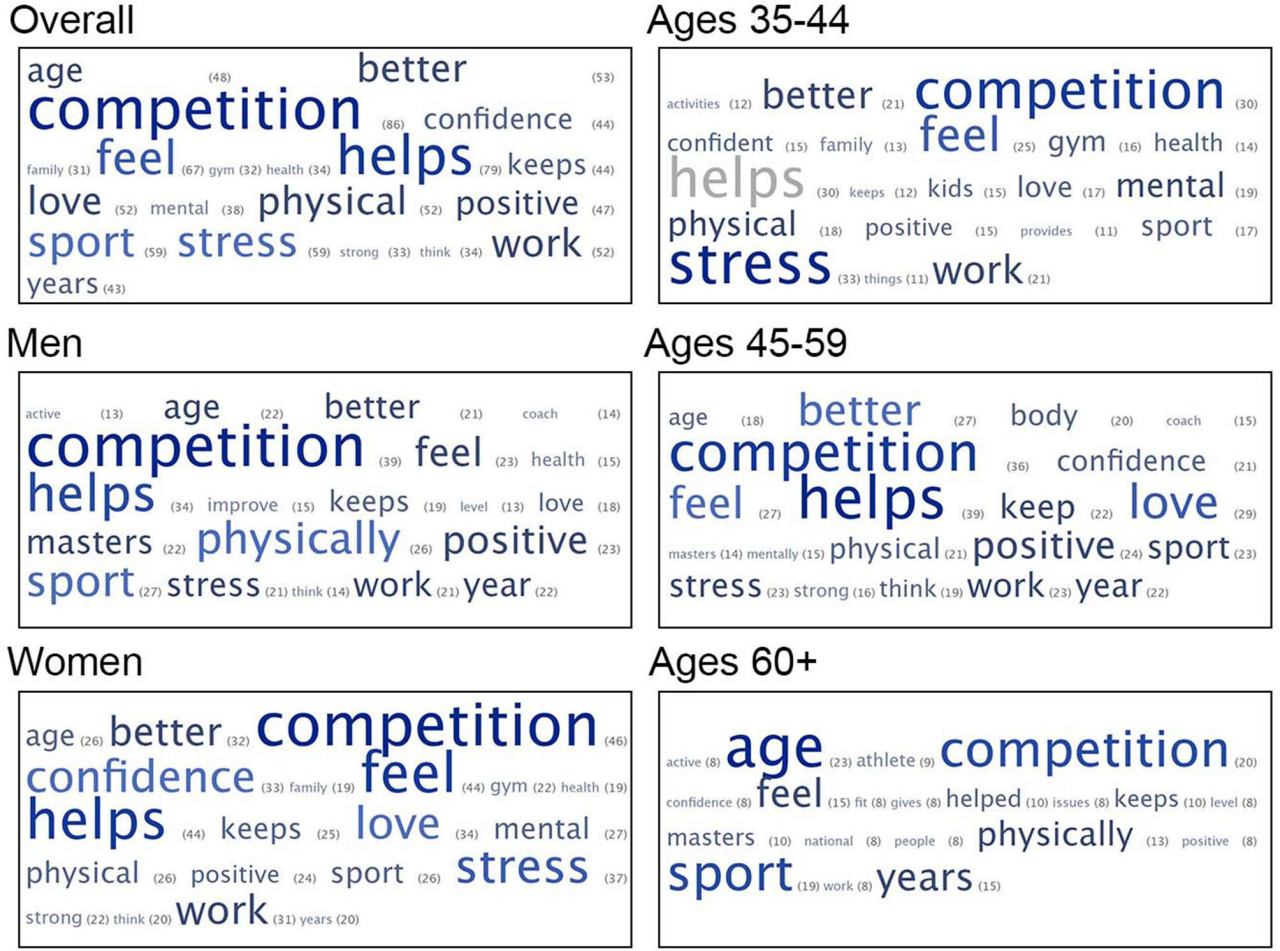
Word clouds showing relative frequency of individual words in participants' comments (counts in parentheses). More frequent words are larger.

In parallel with other analyses in this manuscript, we created five different participant groups based on age and gender and generated a unique word cloud for each group. This comprised all females (*N* = 191), all males (*N* = 159), and three age groups 30–44 (*N* = 138), 45–59 (*N* = 142), and 60 or older (*N* = 74). The most prominent words across gender and the three age groups were “competition” (which grouped together variants such as “compete” and “competes”) and “helps” (Figure 4).

### Age Differences

In the categories from the content analysis, older athletes commented more about general physical health, with odds ratio 1.14 (95% CI: 1.03, 1.27, *p* = 0.012) for each 5-year increase in age (Table 1). Men varied across age group for the general physical health and fitness category, with comments least common in the youngest age group (17%) and more common in the middle (35.9%) and oldest (31.2%) age group, although it did not reach statistical significance at the 0.05 level [χ^2^(2) =4.90, *p* = 0.086) (Figure 1). This corresponds to the results from LIWC analysis where the frequency of health words increases with age [*t*(347) = 2.59, *p* = 0.010] (Table 2). The physical health aspects remained significant even after adjusting for case weights (Supplementary Table 3 for content coding and Supplementary Table 4 for LIWC).

**Table 1:** Odds ratios (OR) and confidence intervals (CI) from logistic regression models for comments coded in different content categories.

**Content categories**		**OR**	**95% CI**	***P***
General physical health	Age	1.14	(1.03, 1.27)	0.012
	Male	0.77	(0.48, 1.24)	0.289
Specific health	Age	1.13	(0.96, 1.34)	0.141
	Male	0.95	(0.44, 2.05)	0.887
Lifestyle health habits	Age	0.95	(0.79, 1.14)	0.560
	Male	1.04	(0.47, 2.32)	0.915
Psychology/Mental health	AGE	0.92	(0.83, 1.02)	0.119
	Male	0.48	(0.31, 0.74)	0.001
Meaning, purpose, goals	Age	0.99	(0.89, 1.1)	0.823
	Male	1.06	(0.66, 1.71)	0.803
Family or community	Age	0.99	(0.88, 1.1)	0.800
	Male	0.71	(0.44, 1.14)	0.155
Commitments required	Age	0.84	(0.71, 1.01)	0.058
	Male	0.78	(0.39, 1.58)	0.494
Work	Age	0.85	(0.72, 1.01)	0.065
	Male	1.19	(0.61, 2.32)	0.616

*Age is in 5-year increments*.

*Odds ratios above 1 for age indicates that comments in the category were more common with increasing age; odds ratios below 1 for male means that comments were more common among women compared to men, odds ratios above 1 for male means that comments were less common among women compared to men*.

**Table 2:** Estimated coefficients for age and gender associated with LIWC categories from a linear regression model.

**LIWC categories**		**Estimate (SE)**	**T-statistic**	***P***
Affective processes	Age	−0.16 (0.21)	−0.76	0.445
	Male	−1.12 (0.95)	−1.19	0.236
Positive emotion	Age	0.09 (0.19)	0.51	0.611
	Male	−0.19 (0.82)	−0.23	0.816
Negative emotion	Age	−0.26 (0.1)	−2.57	0.011
	Male	−0.85 (0.45)	−1.89	0.059
Social processes	Age	0.03 (0.13)	0.27	0.789
	Male	−0.87 (0.56)	−1.56	0.119
Biological processes	Age	0.28 (0.12)	2.29	0.023
	Male	−0.14 (0.55)	−0.26	0.797
Health	Age	0.28 (0.12)	2.29	0.023
	Male	0.29 (0.11)	2.59	0.010
Drives	Age	0.29 (0.2)	1.48	0.141
	Male	0.46 (0.49)	0.94	0.347
Work	Age	0.07 (0.10)	0.71	0.476
	Male	−0.05 (0.45)	−0.10	0.919

Comparing word clouds across the age groups (Figure 4), the most obvious change over time is the prominence of the word “age,” which is the number-one word for the oldest age category (60+), sufficiently infrequent to be absent in the youngest group, and at a relatively low frequency in the middle group. Thematically similar to age, “Year/Years” also becomes more prominent in the older age groups. Words that appear in the younger and middle-level masters but disappear in the oldest group are “Mental/Mentally,” “Stress,” and “Work.”

### Gender Differences

Men were less likely than women to make comments coded under psychological impact (30.8 vs. 51.3%) (Figure 1) with odds ratio 0.48 (95% CI: 0.31, 0.74, *p* < 0.001) (Table 1, Supplementary Figure 3). Women mentioned specific health issues most commonly in the age range 45–59 (12.8%) compared to younger (3.4%) or older ages (8.3%) [χ^2^(2) =5.1382, *p* = 0.077]. This is an age range that includes perimenopause and early menopause for most women. Estimates from the weighted regression analyses were comparable (Supplementary Table 3).

Computer coding classified men's comments less frequently than women as negative emotions [*t*(347) = −1.89, *p* = 0.059) (Table 2; Supplementary Table 4). This was particularly the case at younger ages for women, with negative emotion highest in the 35–44 age group (3.6 vs. 1.9 and 1.4%, Figure 2) which can also be seen by the non-overlapping confidence intervals (Figure 3). This corresponds to the higher frequency of female comments about negative impacts noted in the content coding. Comments with the negative word “sacrifice” include “*There are sacrifices for sure, time being the biggest one*” (Female, 40a), and “*I sacrifice a lot of time away from home in order to train”* (Female, 50s). However, some of the comments that include multiple negative emotion words according to the LIWC dictionary (words such as stress, anxiety, or depression), were actually about positive impacts, which is only evident in the full context of the comments: “*It helps tremendously with stress, anxiety, and depression”* (Female 40s) or “*It is my biggest stress relief”* (Female, 30s). For men (but not women), the incidence of words coded as biological and health increased monotonically across the age groups [*t*(157) = 3.06, *p* = 0.003 and *t*(157) = 3.28, *p* = 0.010, subgroup analyses for men]. Note that health is a subcategory of the biological processes library in LIWC; a comparison of the percentages suggests that health words account for almost all of the biological processes difference.

Comparing the word clouds for two genders collapsed across age categories we see some gender differentiation in the medium frequency words (Figure 4). “Confidence” is quite prominent for women and “Mental” relatively frequent; both words were infrequent enough among males that they do not appear in the men's cloud. For men, the only prominent word that did not make the cut in the women's cloud is “Masters.” Among the words that appear as prominent across the two genders, we can see some variance in emphasis. “Physically” and “Sport” are more prominent for men; “Love” and “Stress” are more prominent for women.

## Discussion

The different approaches (holistic coding by human raters vs. computer-tallied frequency counts) and different levels of analysis (full quotes; words that fit different domains; individual words) help highlight findings that showed up across multiple views of the data. Following the components of the socio-ecological framework, we first discuss personal characteristics and individual behavior, followed by the social/cultural component. We address the environment/policy implications last.

### Positive Impacts on Fitness and Other Health Behaviors, Especially for men

A prominent intrapersonal impact in the data set was the contribution to physical fitness and health. Close to 30% of respondents made comments coded under overall physical health/fitness, and about 15%_made comments coded as specific health or health habits. Respondents credited weightlifting with making them strong, improving flexibility, keeping them generally fit and healthy, and helping with specific health challenges by reducing back pain, increasing bone density, or coping with cancer surgery. Our analyses did indicate some moderation by age and gender, with men showing a stronger emphasis on health/fitness in the older age categories according to the content coding, using more health words than women in the LIWC analysis, and using the words “physical/physically” more commonly than women in the word cloud.

Participants commented on how the strength and fitness gained through weightlifting helped them avoid injury, cope with health challenges, and make them more capable in everyday activities, showing the spillover effects from one domain of individual behavior to other activities, as illustrated by this male lifter in his 50s: “*My weightlifting has enhanced my life, it allows me to change tires, chop trees or any other physical activity I choose to partake. I look at weightlifting as an insurance policy, I pay the premiums in the gym and the work I complete helps me stay strong and lessens my risk of injury*” or for this male lifter in his 70s: “*Brain function and memory have improved to a surprising level. Cognition and information processing seems in line with when I was a young man*.”

Along with expanding the capacity to handle physical challenges safely and easily beyond weightlifting, some respondents discussed how their sport helped them reduce or eliminate negative activities. Some of the lifestyle/health benefits mentioned included eating better, losing excess weight, getting more sleep, and quitting smoking or drinking. A male athlete in his 30s reported that “*It pulled me out of the grips of my own alcohol abuse. I was a lost soul for a long time until I discovered the Snatch and Clean & Jerk.”* And from a female athlete in her 30s: “*Weightlifting changed my life. I quit drinking and smoking.”*

The physical health and fitness theme aligns with a review of articles on aging and competitive sport, which found physical fitness to be a primary motivation for sports participation in quantitative studies of athletes in their 40s and 50s and also in quantitative and qualitative studies of men and women in the age range 55–90 (Dionigi, 2006). More recent work on male and female sports competitors affirms this theme as well (Heo et al., 2013; Jenkin et al., 2017; Stenner et al., 2020). Prior qualitative studies of Masters athletes have not, to our knowledge, highlighted stories of overcoming addiction, however. This may be because an anonymous survey response seems a safer place to share such stories than interviews with researchers or focus group discussions with teammates.

### Positive Psychological Impacts Are Especially Prominent Among Women

At the intrapersonal level, psychological impact comments had the overall highest frequency in content coding and the category with the clearest gender difference. Women had a significantly higher frequency of comments than men in the content coding (51.3 vs. 30.8%), and proportionately more emotion words in the LIWC analysis, and used the word “stress” in their comments more commonly than men.

The three most common mental health issues mentioned all appear in this quote: “*It helps tremendously with stress, anxiety, and depression”* (Female, 40s). Among USAW Masters lifters, the female/male ratio of lifters reporting depression and/or anxiety in the past 2 years was 1.8/1 (Huebner et al., 2020), in line with well-established gender difference in diagnoses of depression (Albert, 2015; Lusa and Huebner, 2021) and anxiety (McLean et al., 2011), with female/male ratios around 1.7/1 for both. Helping weightlifters cope with these challenges is an important benefit of the sport, and aligns with evidence that Masters athletes report a higher level of psychological functioning relative to normative data (Bardhoshi et al., 2016) and that strength training in general provides psychological benefits (Moraes et al., 2020). Young and colleagues also found that among Masters swimmers, “stress relief” was mentioned more by women than men (Young et al., 2015). The dual role that depression/anxiety/stress play impact the training of Masters lifters (Huebner et al., 2020) while also being mitigated by that same training is a nice example of bidirectional influences at the intrapersonal level.

The promise of sports and strength training in helping older adults cope with stress, anxiety, and depression may be useful in encouraging more participation, especially of women. However, it is important to note that men who have such challenges are similarly appreciative of how weightlifting has helped them, as illustrated by the following quote: “*I am diagnosed with PTSD and Depression […]. Since I started Weightlifting, my mental health has improved so much. I went from having daily episodes of deep emotional distress prior to Weightlifting, to now where I have moments of sadness or something come into my life, as will happen, and I don't spiral into a deep depression. Said another way, I have more peace in my life since I started Weightlifting than I think I ever had. I think there are many factors that have resulted in this marked improvement in my mental health, but Weightlifting is one of the bigger factors.”* (Male, 30s)

Along with helping mitigate negative psychological issues, weightlifting was also credited with boosting psychological positives such as self-confidence and mental toughness, and such endorsements were more common among women than men. The gender difference may result from the extra confidence boost that women get from simultaneously countering two stereotypes—the association of both age and female gender with weakness (Ellemers, 2018; Jin and Harvey, 2020). As one female lifter in her 50s commented, “*Throwing around heavy weights safely at this age is an amazing confidence builder.”* For men, participating in a strength sport aligns with traditional constructions of masculinity (Drummond, 2008), so the primary stereotype they are pushing against is the association of old age with weakness. This may explain why the much less common “confidence” statements by men were in the 70+ age group and were paired with “physical/physically” words: “*Satisfaction that I can do it. Physical confidence”* and “*It has benefited my confidence mentally as well as physically.”*

### Competition and Community: The Intrapersonal/Interpersonal Interface

Competition and related themes of goals and achievement are evident across gender identity and age. In the content coding, a little over a quarter of participants (26%) made comments in the “goals/meaning in life” category, which included references to competitive goals and identity as athletes. Variants of the words “compete/competes/competition” dominate the word clouds across age and gender, and “sport” is also prominent. In the LIWC analysis, 12.3% of all words in the comments appeared in the “Drives” library, which includes words related to affiliation, achievement (win, success), and reward. This aligns with previous findings that competing and the associated feelings of achievement are an important reason for older adults to participate in sports (Stenner et al., 2020) and that competitiveness is highly valued among both male and female athletes participating in the Masters Games (Dionigi, 2006; Dionigi et al., 2011). Although Young and colleagues reported that among Masters swimmers, men emphasized competitive achievements more than women (Young et al., 2015), no gender differences were apparent in our data.

From the social ecological perspective, the larger social structure of competition, in which athletes come together to perform the technically challenging lifts they have been practicing during training, is where the individual behavior of training and the social interaction with one's coach (for those who work with a coach) intersect with a larger community of weightlifters. The calendar of upcoming competitions helps structure the individual behavior of training, as noted by two weightlifters in their 50s, a male: “*[My]*_*weightlifting career... gives me, at an older age, goals and daily structure”* and a female: “*Competing gives me something to look forward to set goals for and feel proud of*. The structure of competition also creates a community of like-minded athletes who train together and support one another, and this explicitly social benefit is something that respondents appreciate. A male lifter in his 40s commented: “*It is great to be part of a team again - my first since high school sports. It provides a great community that isn't my job or my family.”*

Documenting that people who chose to devote substantial time and energy to competitive sports value competition is not surprising since the study participants were competitive athletes. This was consistent across gender and age. In an international study of weightlifters in Australia, Canada, Europe, and the United States, that assessed motivation for participation in weightlifting, over 90% selected “agree” or “strongly agree” for the opportunity to compete (Huebner et al., 2021a). As studies in the sociology of sport note (e.g., Dionigi, 2006; Dionigi et al., 2013), competitions for older athletes have tended to embrace themes of “friendship, fun, and fitness,” apparently viewing the “faster, higher, stronger” theme of the Olympics and other competitions for younger adults as less appropriate in later years. However, empirical studies of older athletes (Dionigi and O'Flynn, 2007; Dionigi et al., 2011), consistently find that competitiveness as a motive has not evaporated with age, belying (or actively resisting) the stereotype. As Dionigi and Flynn note, “the competitive older athletes in this study are refusing what the dominant view informs them that they are or should be” (Dionigi and O'Flynn, 2007). This is also true for our sample of Masters age weightlifters, who tend to integrate appreciation for competition as a source of challenging goals with other benefits such as fitness and self-confidence.

In an extensive set of interviews conducted with athletes aged 55–94 (Dionigi, 2006), found that participants in the Australian Masters Games integrated the themes of “competing to win” and “friendship and fun” by being seriously competitive on the field, but friendly and social off the field. For some of the respondents the community aspect has become a major part of their support system beyond the lifting platform, as noted by a female lifter in her 30s who was also a graduate student: “*Weightlifting and the community it has exposed me to are one of the TOP reasons I made it through my PhD. The distraction, the peer support, hell even three of my teammates came to my defense. It is such an incredible community”* or by a transgender lifter in their 60s: “*The sport, travel, friends are an essential part of my reason for staying involved.”* Training in mixed age groups also can provide a “family” feel to the weightlifting community as in this quote by a male in his 60s: “*I train with young people which keeps me young. Love the community.”*

### All in the Family…. or Not: Intersecting Communities

Family responsibilities and athletic training and competition were well-integrated for some respondents, but not all, with the impact of their weightlifting careers having both positive and negative impacts. In line with the social ecological perspective, the intersection of family life with weightlifting life (two microsystems) creates multiple influences that sometimes harmonize and sometimes conflict.

Prior literature (e.g., Dionigi et al., 2012) has tended to emphasize the stress of managing family responsibilities while training. Along with some comments about balancing the two: “*I don't train on weekends. Weekends are for home and family”* (Male, 50s), there were several comments about weightlifting being a shared family activity: “*Being able to train together as a family gives us something else in common that we look forward to and enjoy together”* (Male, 40s).

Comments indicating that conflict with family and relationship obligations were themselves a source of stress came primarily (although not exclusively) from women: “*It is sometimes hard to not feel selfish/guilty for devoting so much time to training that I could be spending with my kids or fiancé.”* (Female, 40s), and marital conflict, which has also been identified by Horton et al. (2018), came up in a few comments: “*Weightlifting puts a strain on my marriage. My husband does not approve of the time, money, or aesthetics”* (Female, 50s) and “*My wife tends to give me a hard time if I go to the gym “too often” or compete on an occasional weekend”* (Male, 40s).

### The Cultural Macrosystem: Grappling With Aging

Although it was not a category in either the content coding nor the LIWC, aging showed up as an increasingly prominent theme in the word clouds, in the words “age” and “year/s.” In fact, age is the most prominent word for the oldest age group, edging out “competition.” This aligns with the finding of Young and colleagues that for Masters swimmers (Young et al., 2015), the opportunity to counter both the physical process of aging and negative stereotypes of older adults becomes more important with increasing age.

The literature on sports participation in older adults has investigated how older athletes view aging, using the interpretative frameworks of both the traditional constructions of aging as a process of increasing disability, disengagement and dependence and the counter discourse of healthy or positive aging (Dionigi and O'Flynn, 2007). Older athletes tend to reject the first and affirm the second. A more recent narrative apparent in conversations with some older athletes (Gard et al., 2017) can be viewed as a moralizing extension of the “healthy aging” construction that views less active older adults as morally inferior or lazy. Athletes tend to reflect their views of aging *via* social comparisons—to other people and to themselves at a different age. In our data, we detected all three strands of discourse.

An example of making fun of traditional constructions came from a female lifter aged 60+: “*Most friends and family find it odd to take up lifting at such an “advanced” age*. *Haha.”* The view of sport as a way to counteract or transcend age came from a male lifter aged 70+: “*It gives me feeling that I live a full life, almost the same, as when I was young, in my family and society*.” An example that blends the “transcending age” with the “moralizing” view of older athletes as superior came from a female lifter aged 60+: “*And my weightlifting career definitely boosts my energy and self-esteem and self-confidence. It keeps me from feeling older. I'm getting better, while others around me just AGE*.”

From a social ecological perspective on context, athletes are not simply reflecting available views on aging—they are also actively contributing to evolving norms and cultural conceptions, by affirming, rejecting, or exploring different meanings of themselves as Masters athletes. This is most evident in the rejection of stereotypes and the quest to enact new images of what it might mean to be old, in some cases by embracing the identity of a role model. Both male and female lifters mentioned this as an impact of their weightlifting career on their role as parents. A father in his 30s commented that “*[It] shows my kids that you can do anything at any age possible as long as you give it your best effort”* and a mother in her 50s reported that: “*Weightlifting and competing has also shown my three*_*daughters that no matter how old you are, you can still find a sport that challenges you!”* Another mother in her 30s framed her role modeling as countering both age and gender stereotypes: ‘*Helps to show my young daughter that it is ok to be a woman and to be strong and powerful.”*

Conflicting views of “age-appropriate” behavior can also create social conflict, showing how the cultural component of norms is contested at the interpersonal level as different perceptions clash. A female lifter in her 40s reflected on how incompatible perspectives put strains on her friendships. Instead of being viewed as a role model, this Masters athlete was simply be viewed as inexplicably odd: “*Socially, it is hard to make people understand the desire to compete at my age. I have lost many friendships because I chose to train and eat healthy consistently, as opposed to on a whim, and I can't drink alcohol as often as my peers do and many of them found that to be, and I quote, ‘weird.”’* Another female lifter, also in her 40s, avoided this sort of conflict because her circle of friends was embedded in her weightlifting community and hence likely shared her orientation toward aging: “*The weightlifting community is remarkable, I have the best friends of my life because of weightlifting and the physical/mental/emotional benefits are unmatched by anything else in my life.”*

### Strengths and Limitations

This study focuses on the analysis of one open ended question within a survey of Masters weight-lifters and thus does not fit in the qualitative research category (Pluye and Hong, 2014). Instead it takes a quantitative approach to extracting meaning from optional comments offered by over a third of those who completed the larger closed-form survey (Huebner et al., 2020). Although it appears that respondents were demographically similar to non-respondents except for being somewhat older and more educated, we do not know to what degree the nature of non-respondent's thoughts might have differed. The strongly positive tone of comments (which exceeds norms established for the LIWC for expressive writing) might result in part from a self-selection effect, with the more enthusiastic devotees of the sport being more eager to share their thoughts. Thus, the percentages in the current study should be viewed as a reflection of what respondents were most motivated to talk about. This is both a limitation and a strength as participants were not constrained by questions and prior interpretative frame chosen by interviewers.

Our population shares the limitations of most studies of older adults in competitive sports by being primarily white and highly educated. We sampled from the entire population of Masters weightlifters registered with USA Weightlifting and thus were able to include a broader population than studies of participants at the World Masters Games, which are limited to those who have both the inclination and resources (time, access to training facilities, adequate wealth, and ability to travel) to participate in a leisure activity that is much less accessible to those with fewer resources.

Strengths of the study are the large sample size, the wide range of ages, a good balance of male and female weightlifters plus two respondents of a different gender, and the rich database of free form responses. The analysis was conducted in three different ways and similar themes emerged across the qualitative content coding, the LIWC analyses, and the word clouds.

Most research studies in the realm of psychosocial effects of sport on older adults focus on smaller age ranges [e.g., over 65– (Eman, 2012), or in their 70s - (Grant, 2001)], or study men and women separately (Drummond, 2008; Dionigi et al., 2011; Horton et al., 2018, 2019), and use convenience samples such as participants in the World Masters Games. Our study adds to the existing literature by comparing comments by gender and among a wide range of age from middle age to older adults within a single sample from the same larger population of Masters weightlifters. It affirms the continuity of themes across ages and genders but also highlights some differences. This is a strength compared to studies that compare athletes of different ages but draw the two age groups from different sports (Litchfield and Dionigi, 2012) or reviews that are restricted to a relatively narrow age range; for example, studies with athletes aged 65+ (Gayman et al., 2017). Some authors are currently expanding their inquiries to other countries, which will help provide a better picture of possible cross-national differences (Huebner et al., 2021a).

## Conclusions and Future Directions

Our cross-sectional study of Masters weightlifters shows that many benefits can be derived from competitive weightlifting for older athletes who are motivated to improve or maintain physical health, fitness, and strength. Perceived benefits include physical fitness, management of stress and other psychological challenges, or injury prevention. Setting goals and the opportunity to compete was evident in the comments. While these themes were identified from responses to an open-ended question at the end of a quantitative survey, they align with findings from small qualitative studies in other Masters sports suggesting that there are common benefits for older athletes regardless of the type of sport. Due to our large sample, we are able to contribute quantitative comparisons for gender and age differences.

The main findings are (1) All age groups reported strongly positive psychological benefits of both training and competition, with setting and achievement of competitive goals providing great motivation for training; (2) Younger Masters athletes emphasized issues related to work and stress, while older athletes had a relatively greater focus on aging and general physical health and fitness; (3) Female athletes commented more frequently than men about the psychological impact of training and competition, often mentioning increased confidence as a significant outcome; (4) Stress relief was more commonly mentioned by females than males.

Participants in our study expressed pride in their achievements in comparison to other people at similar ages, indicative of the benefits experienced by Masters athletes who, among other challenges, must overcome stereotypes of successful aging. For example, in the past, Masters weightlifters have often been neglected or excluded by weightlifting's governing bodies. For years after the first World Masters Weightlifting Championships were held in 1985, the International Weightlifting Federation (IWF), the world governing body for Olympic weightlifting, rejected attempts by the Masters to receive official recognition. As late as 1991, IWF was still withholding their formal endorsement, publishing a widely circulated statement titled “We Should Not Disturb Them” (World Weightlifting, 1991/2). However, after a lengthy struggle, Masters were formally recognized by the IWF in 1993 with the establishment of the IWF Masters Committee. USA Masters Weightlifting is both self-organized and integrated in USAW, the national governing body that sanctions competitions for all ages and performance levels.

Women's participation in competitive sports, in particular in strength sports, was initially controversial, although women first started competing in weightlifting in the 1940s (Strength & Health, April 1947, pp. 24–25, 33–34) and then again in the 1970s (Glenney, 2020, p. 122). This experience was described by a Female in her late 50s: “*I'm in awe of what females in our sport can do today and the opportunities they enjoy. Fantastic! When I start[ed] lifting, women were not allowed to compete. Evidently, I was allowed to compete with men for a year or two, I had to lift in male weight classes and use equipment designed for men. It wasn't easy...”* Participation of women in age groups older than 65–69 is still sparse; this group includes women who grew up during the 1970s, whose average age for starting weightlifting is 60. Thus, weightlifting stereotypes are related to both age and being female. Ageism is experienced by older athletes in the fitness industry in general (Jin and Harvey, 2020). Fitness centers have strength equipment, but typically only carry the heavier barbell used by men in a competition.

The environment/policy level of the socio-ecological framework (King and King, 2010) focuses on community-level policies and programs and policies of governing bodies. While the survey question was about how weightlifting affects other parts of life, from the responses we were able to identify competitions and educational opportunities as themes.

### Implications for Communities and Coaches

Weightlifting is an individual sport and is suitable for training in several venues, including at home. However, access to equipment and training with high loads of weight presents a unique problem to this group (Latella and Haff, 2020). Clubs may focus on developing younger athletes, and, due to restricted time of access, Masters athletes may need to find training opportunities at different locations including fitness centers, CrossFit boxes, facilities at work, or at home. A substantial proportion (25–40%) of weightlifters utilize multiple training locations (Huebner et al., 2020, 2021a). Weightlifting has been termed the “underground sport,” as it so often takes place in basements of homes or public facilities. Survey respondents reported a diverse set of experiences at their training locations, some having trained in communities inclusive of older adults while others felt more isolated as evidenced by a male lifter in his 70s: “*Our gym is a very welcoming village that had added a new dimension to my life”* or by a female lifter in her 30s” “*Being in a smaller (not urban areas) has limited some factors... Access to gyms with Olympic lifting equipment, no local coaches, required travel/overnights to attend meets, and limited community to lift with”* More guidance is needed regarding how to promote diversity and inclusivity in clubs and communities.

Coaches and Masters athletes are often left to make their own decisions about what is appropriate for older athletes to perform and grapple with different sources of information, including ones that may not be appropriate for Masters sports or for their individual situation. Coaching Masters athletes may require different approaches than coaching younger athletes (Young et al., 2014; MacLellan et al., 2019; Hoffmann et al., 2020; Currie et al., 2021). Coaching professionals who intend to include competitive weightlifters among their clients would benefit by being aware of these findings, enabling them to respond proactively to their clients' needs. More research is needed to understand training regimens for older athletes or different genders.

### Implications for Sport Organizations

Sport organizations can support Masters athletes through education of athletes and coaches, organization of competitions, promoting access at regional levels, and policies of inclusivity (Medic et al., 2019; Callary et al., 2021 and references therein). Recently, USAW has taken steps to enhance diversity and inclusion related to LGBTQIA+ and BIPOC communities and to drive change by restructuring Weightlifting State Organizations (“USA Weightlifting Taking Further Steps To Ensure The Sport Is Accessible, Safe And Welcoming To ‘Anyone, Anywhere”’ 18. Nov. 2021) (Team, 2021).

The desire to participate in USAW training camps for Masters athletes with high quality coaching was expressed by some participants *("It has been a great opportunity to train with many high level athletes, including Olympians, train at the first masters camp in COS [Colorado Olympic Training Center] and also many high level coaches, who also were Olympic coaches”)*. New national and international efforts have started with the COVID pandemic in 2020 including the organization of virtual weightlifting competitions and the offering of educational webinars. Virtual weightlifting competitions with live streams and real-time refereeing mirrored in-person competitions as closely as possible. An international survey of weightlifters indicated a desire that such events continue as qualifying events, but not necessarily the national or international championships, due, for example, to the lack of doping control in virtual events. These sentiments were expressed by 60% of the women and 40% of the men (Huebner et al., 2021a). Masters sport participants are often characterized as affluent and highly educated (Wright and Perricelli, 2008; Huebner et al., 2020) and such online events have the potential to lower barriers for athletes through reduced cost and time for travel as identified by several participants in the survey.

The organization of regional competitions can also provide challenges in finding an affordable venue and in the need to transport a large amount of equipment, to set up warm-up platforms and technology equipment at the venue. Masters weightlifters volunteer in administrative roles, are engaged in the sport as coaches, and serve as technical officials at all levels of competitions, and thus are a great resource for sport organizations. All levels and ages are typically welcome at regional competitions (local and state championships) but also at national levels (American Open Series) where competitors range in age from under 10 to over 80 years. Competitions heavily rely on volunteers with some positions requiring certification (e.g., referees) while other positions are open to all (e.g., loaders of the barbell). Older and younger athletes help each other and may even lift in the same sessions. These are excellent opportunities for interactions and to learn from each other (Fairchild, 2019). Older athletes can function not only as role models for the younger athletes, but also as good examples for families who constitute part of the audience at these events. Masters weightlifters mentioned their great enjoyment at meeting other older athletes at competitions that are organized for, and specific to, Masters. Sport organizations can provide such opportunities by sponsoring competitive events for older athletes (Kirby and Kluge, 2013; Huebner et al., 2021a).

Although we found a great deal of continuity across age groups, it is likely that the relative importance of Masters sport for different aspects of life—physical and mental health, community and competition—may shift over time, and may also differ for different populations of athletes including those who are returning to sport after a long break, those who have been continuously active in sport for most of their lives, and those who took up a competitive sport at an older age. Thus, following older adults in a longitudinal study to reflect on age-related changes could be a step toward learning about the impact of training and competing across the lifespan and can help refine or knowledge of the long-term outcomes of such activities.

## Data Availability Statement

The datasets presented in this article are not readily available because qualitative data are presented in aggregate due to the possibility of identifiability of raw data. Requests to access the datasets should be directed to huebner@msu.edu.

## Ethics Statement

The studies involving human participants were reviewed and approved by Institutional Review Board of Michigan State University (Study No. 00003824). The patients/participants provided their written informed consent to participate in this study.

## Author Contributions

MH and HA contributed to the concept. HA and AG performed the content coding and selected quotes. MH and AG performed the data analysis. MH, HA, and DM interpreted the results, and wrote the first draft of the manuscript. All authors approved the final manuscript.

## Conflict of Interest

The authors declare that the research was conducted in the absence of any commercial or financial relationships that could be construed as a potential conflict of interest.

## Publisher's Note

All claims expressed in this article are solely those of the authors and do not necessarily represent those of their affiliated organizations, or those of the publisher, the editors and the reviewers. Any product that may be evaluated in this article, or claim that may be made by its manufacturer, is not guaranteed or endorsed by the publisher.
