# Supplementary material for: How Heavy Lifting Lightens Our Lives: Content Analysis of Perceived Outcomes of Masters Weightlifting
Source: Front Sports Act Living. 2022 Mar 15;4:778491. doi: 10.3389/fspor.2022.778491 (PMC8974931; doi:10.3389/fspor.2022.778491)
Supplement: Supplementary file 1 [file Data_Sheet_1.docx]

**How heavy lifting lightens our lives: Perceived psychosocial and health benefits of Masters weightlifting**

Marianne Huebner, Holly Arrow, Alex Garinther, David E. Meltzer

**SUPPLEMENTARY MATERIAL**

**FIGURE S1. Study flow diagram**

**
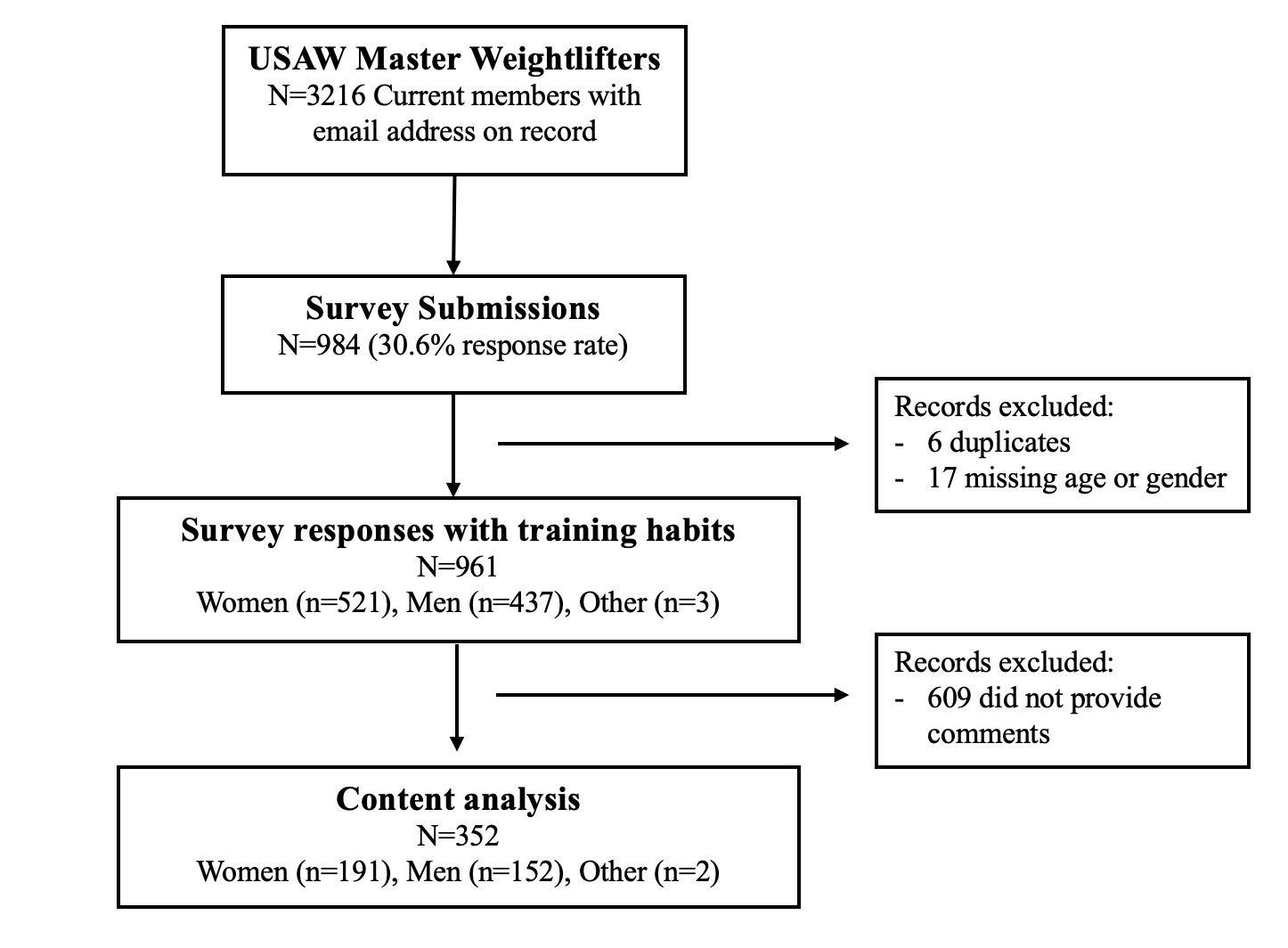
**

**TABLE S1.** Comparison of demographics of respondents providing comments to those respondents not providing comments.

|  | **Survey only (N=609)** | **Survey with comments (N=352)** | **p value** |
| --- | --- | --- | --- |
| **Gender** |  |  | 0.594 |
| Female | 330 (54.2%) | 191 (54.3%) |  |
| Male | 278 (45.6%) | 159 (45.2%) |  |
| Other | 1 (0.2%) | 2 (0.6%) |  |
| **Age;** median (range) | 44 (34-80) | 49 (34-87) | < 0.001 |
| **Hispanic** |  |  | 0.579 |
| N-Missing | 3 | 2 |  |
| Yes | 53 (8.7%) | 27 (7.7%) |  |
| **Race** |  |  | 0.603 |
| N-Missing | 7 | 5 |  |
| White/Caucasian | 492 (81.7%) | 304 (87.6%) |  |
| Asian | 32 (5.3%) | 13 (3.7%) |  |
| Black or African American | 22 (3.7%) | 7 (2.0%) |  |
| Native Hawaiian or Pacific Islander | 6 (1.0%) | 5 (1.4%) |  |
| Other or Mixed | 50 (8.2%) | 18 (5.1%) |  |
| **Education, highest level attained** |  |  | < 0.001 |
| High school or less | 27 (4.5%) | 4 (1.1%) |  |
| Some college | 105 (17.2%) | 38 (10.8%) |  |
| College degree | 215 (35.3%) | 112 (31.8%) |  |
| Graduate school | 252 (43.0%) | 198 (56.2%) |  |

**FIGURE S2.** Odds ratios and 95% confidence intervals for comments coded in different content categories (corresponding numbers are included in Table 1). The vertical line at 1.0 indicates there is no difference. Odds ratios to the left of the vertical line indicate that more women mentioned this than men (left panel) or that it was more common for younger participants (right panel).

**Inverse probability weighting (IPW)**

Since open ended comments were more likely provided by older participants with higher education levels, balancing the demographic factors between responders and non-responders was necessary. For each participant case weights were estimated in a logistic regression model to predict the probability of providing comments. The variables age group, gender, and education are potential confounders and thus were included in estimating the case weights. This approach balances demographic factors that may affect participation. After applying case weights the demographics between these two groups of participants are comparable (Table S2). Estimates from the weighted regression models were comparable to those of the primary analyses.

Weighted multivariable linear models were used to study associations with the computer coded data (LIWC), and weighted multivariable logistic regression models were used to study associations with the content coded outcomes. Similar to unweighted analyses, LIWC negative emotion words were more often used by women and at younger ages (Table S3). Words related to biological processes and health were used by older participants.

Odds ratios and 95% confidence intervals for the IPW models are given in Table S4. Comments on general physical health and specific health are associated with older ages. Women are more likely to mention psychological and mental health factors or family/community. In the weighted analyses some associations are stronger, but the effects are otherwise similar to unweighted analyses.

**TABLE S2.** Comparison of demographics of respondents providing comments to those respondents not providing comments after weighting

|  | **Survey only (N=609)** | **Survey with comments (N=352)** | **p value** |
| --- | --- | --- | --- |
| **Gender** |  |  | 0.984 |
| Female | 54.6% | 54.6% |  |
| Male | 45.4% | 45.3% |  |
| Other | 1 (0.2%) | 2 (0.6%) |  |
| **Age;** median (1^st^, 3^rd^ quartile) | 45 (39, 53) | 46 (39,55) | 0.201 |
| **Hispanic** |  |  | 0.656 |
| Yes | 8.1% | 9.0% |  |
| **Race** |  |  | 0.603 |
| White/Caucasian | 82.5% | 86.5% |  |
| Asian | 5.4% | 4.2% |  |
| Black or African American | 3.7% | 2.1% |  |
| Native Hawaiian or Pacific Islander | 0.9% | 1.5% |  |
| Other or Mixed | 7.5% | 5.7 |  |
| **Education, highest degree** |  |  | 0.991 |
| High school or less | 3.2% | 2.7% |  |
| Some college | 14.9% | 15.1% |  |
| College degree | 39.9% | 40.0% |  |
| Graduate school | 42.0% | 42.1% |  |

**TABLE S3** Odds ratios (OR) and confidence intervals (CI) from weighted logistic regression models for comments coded in different content categories.

| **Content categories** |  | **OR** | **95% CI** | **P-value** |
| --- | --- | --- | --- | --- |
| General Physical Health | age | 1.03 | (1.00, 1.05) | 0.003 |
|  | male | 0.80 | (0.51, 1.23) | 0.313 |
| Specific Health | age | 1.03 | (1.01, 1.06) | 0.003 |
|  | male | 0.89 | (0.41, 1.94) | 0.765 |
| Lifestyle Health Habits | age | 0.99 | (0.96, 1.03) | 0.801 |
|  | male | 1.14 | (0.50, 2.59) | 0.762 |
| Psychology/Mental Health | age | 0.996 | (0.98, 1.01) | 0.713 |
|  | male | 0.55 | (0.37, 0.80) | 0.002 |
| Meaning, Purpose, Goals | age | 1.00 | (0.98, 1.02) | 0.962 |
|  | male | 1.19 | (0.74, 1.89) | 0.473 |
| Family or Community | age | 1.00 | (0.98, 1.02) | 0.706 |
|  | male | 0.61 | (0.38, 0.97) | 0.037 |
| Commitments required | age | 0.97 | (0.94, 1.00) | 0.098 |
|  | male | 0.84 | (0.42, 1.67) | 0.631 |
| Work | age | 0.98 | (0.95, 1.01) | 0.157 |
|  | male | 1.04 | (0.51, 2.10) | 0.913 |

NOTE: Odds ratios above 1 for age indicates that comments in the category were more common with increasing age; odds ratios below 1 for male means that comments were more common among women compared to men.

**TABLE S4.** Estimated coefficients for age and gender associated with LIWC categories from a weighted linear regression model

| **LIWC categories** |  | **Estimate (SE)** | **T-statistic** | **P-value** |
| --- | --- | --- | --- | --- |
| Affective processes | age | -0.02 (0.04) | -0.44 | 0.657 |
|  | male | -1.32 (1.19) | -1.11 | 0.270 |
| Positive emotion | age | 0.03 (0.04) | 0.73 | 0.465 |
|  | male | 0.03 (1.13) | 0.03 | 0.976 |
| Negative emotion | age | -0.05 (0.02) | -2.40 | 0.017 |
|  | male | -1.27 (0.43) | -2.96 | 0.003 |
| Social processes | age | 0.004 (0.03) | 0.15 | 0.878 |
|  | male | -0.78 (0.61) | -1.28 | 0.207 |
| Biological processes | age | 0.07 (0.02) | 2.53 | 0.012 |
|  | male | -0.24 (0.52) | -0.45 | 0.653 |
| Health | age | 0.06 (0.02) | 2.62 | 0.009 |
|  | male | 0.34 (0.45) | 0.881 | 0.379 |
| Drives | age | 0.06 (0.04) | 1.43 | 0.156 |
|  | male | 0.86 (1.12) | 0.45 | 0.447 |
| Work | age | 0.01 (0.02) | 0.76 | 0.451 |
|  | male | 0.20 (0.48) | 0.42 | 0.674 |

**FIGURE S3.** Odds ratios and 95% confidence intervals from weighted logistic regression models for comments coded in different content categories (corresponding numbers are included in Table S4). The vertical line at 1.0 indicates there is no difference. Odds ratios to the left of the vertical line indicate that more women mentioned this than men (left panel) or that it was more common for younger participants (right panel).
